# Descriptive study of cholera-related deaths in communities during Zambia’s 2023–2024 outbreak: key insights

**DOI:** 10.1136/bmjopen-2025-102709

**Published:** 2025-10-21

**Authors:** Oliver Mweso, Agatha Shula, Cephas Sialubanje, Stephen Longa Chanda, Thelma Shinjeka, Kelvin Mwangilwa, Lusungu Chirwa, Danny Kabwe, Jonathan Mwanza, Nyuma Mbewe, Sombo Fwoloshi, Nyambe Sinyange, Muzala Kapina, Azmach Gebregiorgis, Otipo Shikanga, Moses Mwale, Muyereka Nyirenda, Peter Lisulo, Peter Chipimo, Benjamin Mubemba, Nawa Mukumbuta, Nathan Bakyaita, Roma Chilengi

**Affiliations:** 1 Zambia National Public Health Institute, Lusaka, Zambia; 2 World Health Organisation, Lusaka, Zambia; 3 School of Public Health, University of the Witwatersrand Johannesburg Faculty of Health Sciences, Johannesburg, South Africa; 4 University of Zambia School of Medicine, Lusaka, Zambia; 5 Zambia Ministry of Health, Lusaka, Zambia; 6 University Teaching Hospital, Lusaka, Zambia; 7 World Health Organization, Lusaka, Zambia; 8 Department of Wildlife Sciences, The Copperbelt University, Kitwe, Zambia; 9 School of Public Health, Levy Mwanawasa Medical University, Lusaka, Zambia; 10 Public Health, Stellenbosch University, Stellenbosch, South Africa

**Keywords:** Epidemics, Cross-Sectional Studies, Disease Outbreaks, EPIDEMIOLOGY, Public health, PUBLIC HEALTH

## Abstract

**Abstract:**

**Objectives:**

The study sought to understand the characteristics of community deaths due to cholera in Zambia. We sought to examine the drivers of mortality from cholera among communities in Zambia’s 2023–2024 outbreak.

**Design/setting:**

This is a descriptive study of the characteristics of community deaths due to cholera in three provinces in Zambia. Routine surveillance data collected between 14 October 2023 and 16 April 2024, comprising a national line list of cholera deaths, were used for this study.

**Participants:**

178 participants were included in the study and completed it. All community deaths on the line list were eligible for inclusion. This comprised: deceased individuals whose death was associated with cholera or who met the national cholera case definition (suspected or confirmed); death occurring in the community, en route or on arrival to a health facility prior to admission; and death must have occurred between 14 October 2023 and 16 April 2024. Deceased individuals whose family members could not be traced or did not consent to participate in the interview were excluded.

**Primary and secondary outcome measures:**

The primary outcome was identifying characteristics of cholera-related community deaths. There were no secondary outcomes measured.

**Results:**

Among 178 community deaths due to cholera, the majority were males (61.8%), with the highest mortality in adults aged 35–49 years (22.5%). Over half of the deaths occurred on arrival at healthcare facilities due to delays influenced by socioeconomic barriers. Comorbidities such as HIV/AIDS and hypertension were present in 23% of cases.

**Conclusions:**

The study found that males, death on arrival at healthcare facilities, delays in seeking healthcare and comorbidities such as HIV/AIDS and hypertension were more frequently observed among those who died due to cholera in the community. These findings highlight the need for enhanced early care-seeking behaviours, improved access to timely treatment and targeted interventions for individuals with comorbidities to potentially reduce cholera mortality.

**STRENGTHS AND LIMITATIONS OF THIS STUDY**

- All community deaths on the National line list were eligible for inclusion.
- Study participants included both suspected and confirmed cholera deaths who died in the community, en route or on arrival to a healthcare facility prior to admission, ensuring a comprehensive capture of community deaths due to cholera.
- While this study provides valuable insights, it is limited in its study design as it relied on retrospective data, which may be subject to recall bias.
- It is not possible to draw causal inference because of the study design.
- More studies with a prospective and analytical design are needed to draw causal inference.

## Introduction

 Cholera is an acute watery diarrhoeal (AWD) disease caused by ingestion of food or water contaminated with the toxigenic strains of *Vibrio cholerae* serogroups O1 or O139.^1^ Clinical disease is characterised by acute non-bloody watery diarrhoea, severe dehydration and may be accompanied by vomiting.^1 2^ It affects both children and adults and can cause death within hours if left untreated.^2^ Access to safe water, improved sanitation, hygiene promotion, timely and appropriate clinical case management, and oral cholera vaccines (OCVs) are some of the effective control strategies.^2^ Treatment involves, among others: administration of oral rehydration solutions (ORSs), intravenous fluids and/or antibiotics depending on the severity of the disease.^1 2^ The case fatality rate (CFR) for untreated cholera can reach as high as 30%–50%, but with timely rehydration therapy, it can be reduced to as low as 1%.^3^

Globally, cholera is still a major health challenge with an estimated 2.86 million cases and 95 000 deaths annually.^4^ Sub-Saharan African countries bear a disproportionate burden.^5^ Regrettably, the year 2024 witnessed a threatening re-emergence of cholera across the world, with significant outbreaks reported in more than 25 countries. Sub-Saharan Africa recorded the highest number with over 14 countries, including Burundi, Cameroon, Comoros, the Democratic Republic of the Congo, Ethiopia, Kenya, Malawi, Mozambique, Nigeria, South Africa, Tanzania, Uganda, Zambia and Zimbabwe.^6^

Zambia reported the first cholera outbreak during 1977–1978 with subsequent outbreaks in 1993, 1999, 2003–2004, 2005–2006, 2010, 2017–2018 and 2023–2024.^7^ The 2023–2024 outbreak was the largest cholera outbreak in history with clinical cases reaching a peak between October 2023 and April 2024.^8 9^ As of 16April 2024, a total of 22 922 cholera cases and 734 deaths were reported with a CFR of 3.2% and 436 community deaths, three times higher than the WHO’s recommended threshold of less than 1%.^10^ The majority of the reported deaths occurred in the community. Strikingly, Lusaka district was the most affected province, accounting for approximately 85% of all cholera deaths, 59% of which occurred in the community.^9^ Similar trends were observed on the Copperbelt and Central provinces. Persistent and continuous outbreaks of cholera have far-reaching adverse outcomes including diversion of developmental funds, ability to access medical care for the affected individual, threatening the economies of affected communities and ultimately contributing to the cycle of poverty.^11 12^

Given the high incidence of clinical cases and community deaths observed during the outbreak, there is a need to refine public health strategies aimed at averting future outbreaks and reducing community cholera-related mortalities. The present study sought to describe the socioeconomic, environmental and healthcare-related factors associated with the 2023–2024 cholera outbreak. Understanding these factors is crucial for developing policies aimed at protecting at-risk communities against cholera.

## Methods

### Study design

The study was a descriptive design using mortality data from the 2023–2024 cholera outbreak. We reviewed routine surveillance data from the Ministry of Health (MoH) compiled surveillance line list from 14 October 2023 to 16 April 2024, which included cholera deaths.

### Study sites

**Figure 1:**
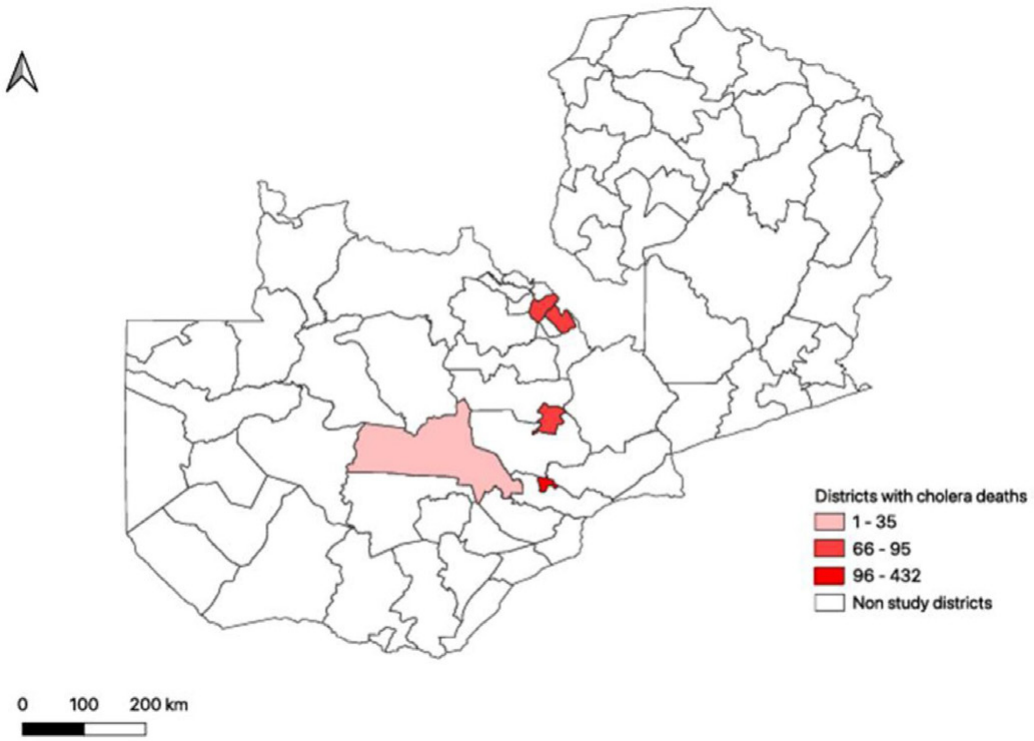
Location of districts that were targeted for sampling of cholera-related community deaths in Zambia’s 2023–2024 outbreak.

The study was conducted in Lusaka, Central and Copperbelt Provinces, focusing on districts that reported the highest number of cholera-related community deaths. These districts included Kanyama, Matero, Munali, Mandevu and Chawama subdistricts of Lusaka province; Kabwe and Mumbwa districts of Central province; and Ndola and Kitwe districts of Copperbelt Province. The districts that were targeted for sampling are shown in figure 1.

### Study population

Study participants included both suspected and confirmed cholera deaths who died in the community, en route or on arrival to a healthcare facility prior to admission, with dates of onset of symptoms or notification between 14 October 2023 and 16 April 2024. The study protocol (available on request) defined suspected cholera death as any deceased person who had AWD (non-bloody liquid stools that may contain mucus) lasting 7 days or less and had at least three or more loose stools within a 24-hour period; whereas, a confirmed cholera death was any deceased person with *Vibrio cholerae* O1 or O139 strains identified by culture/isoagglutination or PCR test.^1 2^

### Sample size calculation

The study used the MoH surveillance line list as a sampling frame and calculated the sample size using a finite population formula .^13^ The formula is n=N/(1+Ne^2^), where n is the sample size, N is the population size, and e is the margin of error.

Assuming a 5% margin of error, with a total number of community deaths from the line list (N=436), the estimated minimum sample size of 209 was calculated.

### Selection of deaths

A random sampling technique was used to select community deaths that were recorded on the MoH surveillance line list. Selection was done using a random number generator.^14^ Thereafter, the selected deaths were then followed up in their respective localities based on their contact details provided on the line list.

Inclusion criteria

- Must have been on the MoH surveillance line list.
- Deceased individuals whose death was associated with cholera or who met the national cholera case definition (suspected or confirmed) occurring in the community, en route or on arrival to a health facility prior to admission.
- Must have died between 14 October 2023 and 16 April 2024.

Exclusion criteria

- Deceased individuals whose family members could not be traced or did not consent to participate in the interview.
- Deceased individuals who did not reside in a cholera outbreak district.

### Data collection and analysis

The data collection team comprised health facility surveillance officers, mortality surveillance officers and trained volunteers. Prior to data collection, the team was trained and equipped on the content of the questionnaire, interview techniques, ethical considerations and the use of electronic devices for data collection.

Data were collected using a structured questionnaire (available in the online supplemental materials). The questionnaire comprised various sections and covered demographic information, clinical symptoms, healthcare-seeking behaviours and environmental factors. Data were collected from relatives or close friends of the deceased cases who had known or interacted with the deceased for at least 7 days prior to their death.

To ensure efficient and accurate data entry, data collection was performed electronically using tablets and smartphones equipped with Kobo Collect software.^15^ Descriptive analyses were performed and presented using frequencies and percentages.

### Patient and public involvement

Participants were not involved in the setting of research priorities, defining research questions and outcome measures, providing input into study design and conduct, dissemination of results and evaluation of studies.

## Results

### Participant recruitment algorithm

**Figure 2:**
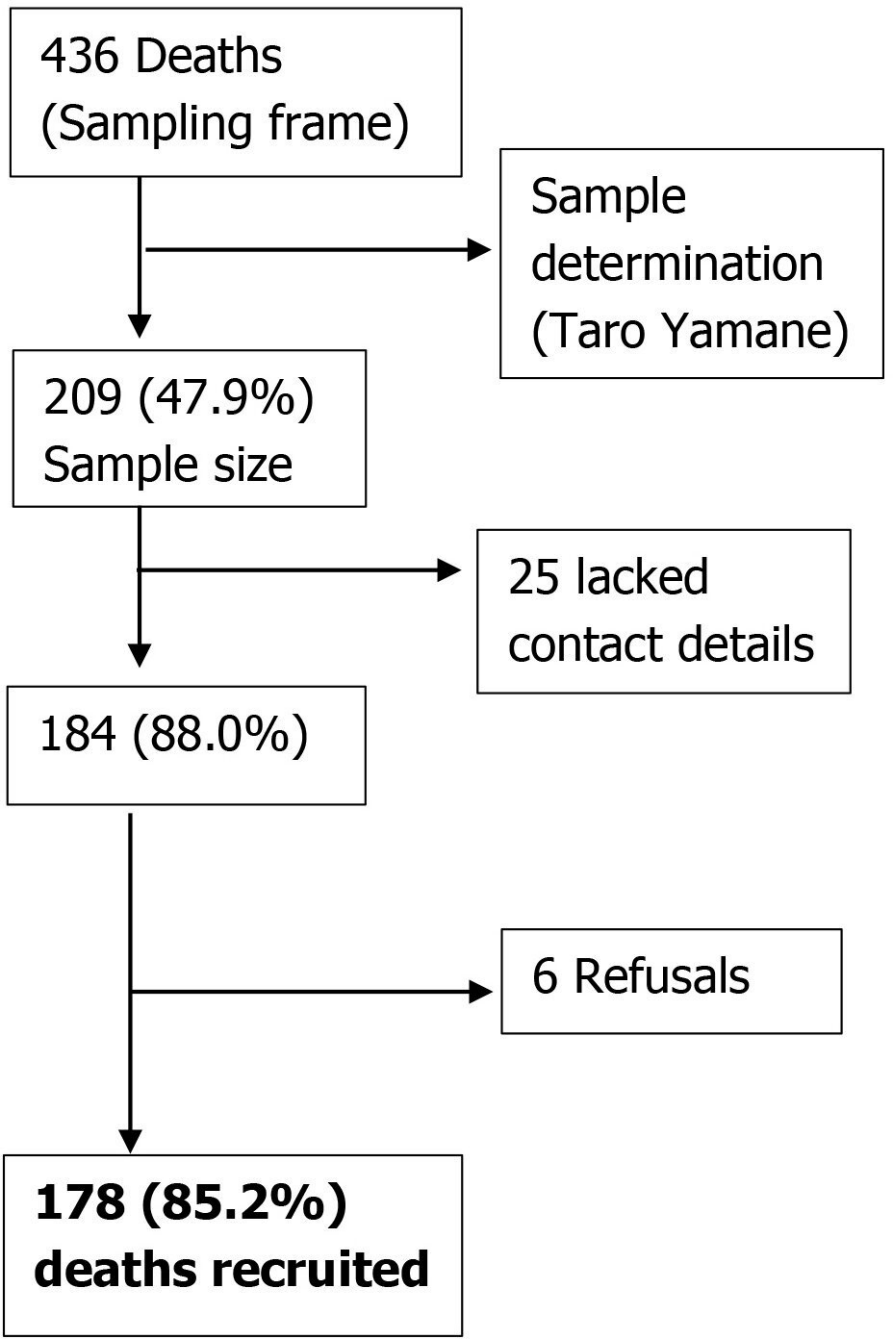
Recruitment flow diagram for cholera-related deaths in communities during Zambia’s 2023–2024 outbreak.

A total of 436 community deaths due to cholera were recorded on the national line list. Using a random number generator, 209 deaths were selected as the sample size. Of these, 25 lacked contact details and could not be followed up, while interviews for six deaths were declined by the relatives. Figure 2 shows the recruitment algorithm of the deceased. A comprehensive analysis was conducted on 178 cholera-related community deaths. The findings are presented under the following categories: demographic characteristics, environmental characteristics, clinical characteristics, vaccination and care-seeking behaviour, patterns in care-seeking behaviour, medication and treatment.

### Demographic characteristics

**Figure 3:**
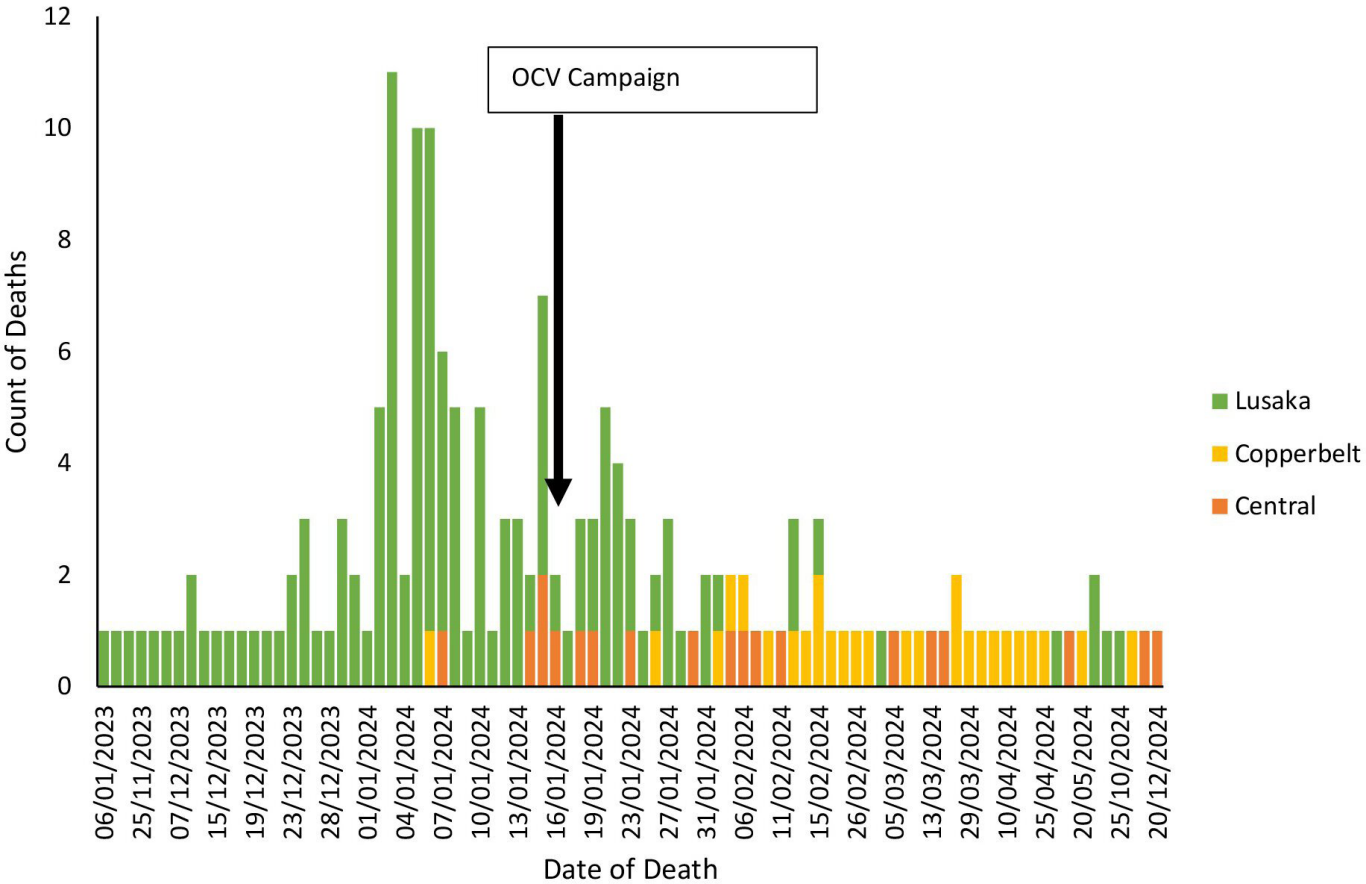
Distribution over time of cholera-related deaths among the three provinces in Zambia’s 2023–2024 outbreak. OCV, oral cholera vaccines.

The majority of deaths were in Lusaka province, with a peak incidence in early January 2024. The distribution of cholera-related community deaths over time in the Copperbelt and Central province was uniform (figure 3).

Males accounted for a higher proportion of deaths (61.8%) compared with females (38.2%). The largest proportions of deaths were among individuals aged 35–49 years (22.5%) and 25–34 years (20.2%); 20.8% were aged above 50 years, 16.3% were in the youngest age group (0–4 years) and 7.3% were in the age group 15–24 years. Approximately 38.2% had completed primary education, 29.2% had secondary education, 24.7% had no formal education and 3.9% had tertiary education. Nearly half (48.3%) of the deceased were single, while 34.8% were married or cohabiting. Family monthly income predominantly fell below 500 Kwacha (US$18): 35.4% earned less than 100 Kwacha (US$3.6) and 18.5% earned between 100 and 499 Kwacha (US$3.6–US$18). Household sizes varied, with households of four or five members being the most common, each constituting 18.5%. Table 1 shows the demographic and socioeconomic characteristics of the deceased.

**Table 1:** Demographic and socioeconomic characteristics of cholera-related deaths in communities during Zambia’s 2023–2024 outbreak (n=178)

Characteristic	N	%
Age group (years)		
0–4	29	16.3
5–14	23	12.9
15–24	13	7.3
25–34	36	20.2
35–49	40	22.5
≥50	37	20.8
Sex		
Male	110	61.8
Female	68	38.2
Educational status		
No formal education	44	24.7
Primary education	68	38.2
Secondary education	52	29.2
Tertiary education	7	3.9
Don't know	7	3.9
Marital status		
Single	86	48.3
Married/cohabiting	62	34.8
Widowed	16	9.0
Divorced	10	5.6
Separated	4	2.2
Family monthly income		
0–99 Kwacha	63	35.4
100–499 Kwacha	33	18.5
500–999 Kwacha	39	21.9
1000–4999 Kwacha	39	21.9
5000–10 000 Kwacha	1	0.6
Prefer not to say	3	1.7
Household size		
1	7	3.9
2	25	14.0
3	17	9.6
4	33	18.5
5	33	18.5
6	15	8.4
7	25	14.0
8	8	4.5
>8	15	8.4

### Environmental and clinical characteristics

Table 2 presents the environmental context of the deaths, and the clinical symptoms reported. Most deaths occurred on arrival at the healthcare facility prior to admission (51.1%) or at home (29.2%). Death during transit from home to a facility accounted for 7.9%, and 3.4% occurred while transferring between facilities. Most deaths occurred in the morning (46.6%); almost one-fifth (18.0%) occurred during the afternoon. Most (90.6%) cases presented with vomiting. Additional symptoms included leg cramps (52.3%), fever (43.4%) and headache (27.2%). Further, comorbid conditions were reported in 23.0% of cases, with HIV/AIDS being the most common (34.2%), followed by hypertension (22.0%).

**Table 2:** Environmental and clinical characteristics (n=178) of cholera-related deaths in communities during Zambia’s 2023–2024 outbreak

Characteristic	N	%
Place of death		
Upon arrival at health facility	91	51.1
Home	52	29.2
In Transit (home to facility)	14	7.9
Other (specify)	13	7.3
In Transit (facility to facility)	6	3.4
Relatives' home	2	1.1
Time of death		
Morning	83	46.6
Night	35	19.7
Afternoon	32	18.0
Evening	26	14.6
Unknown	2	1.1
Symptoms reported		
Vomiting	161	90.6
Leg cramps	85	47.8
Fever	78	43.8
Headache	48	27.0
Comorbid conditions		
None	127	71.3
Yes	41	23.0
Don't know	10	5.6
Types of comorbidities (n=41)		
HIV/AIDS	14	34.2
Hypertension	9	22.0
Others (specify)	8	19.5
Diabetes mellitus	3	7.3
Heart disease	2	4.9
Malaria	2	4.9
Tuberculosis	2	4.9
Hypertension and HIV/AIDS	1	2.4
Alcohol consumption		
Yes	76	42.7
No	102	57.3
Consuming alcohol vs stopped consuming alcohol (n=76)		
Yes*	43	56.6
No	23	30.2
Don’t know	10	13.2
Alcohol withdrawal vs no alcohol withdrawal (n=43)		
Yes†	14	32.6
No	29	67.4

* A deceased individual who stopped consuming alcohol within a week or a month before death.

† A deceased individual with a history of alcohol consumption who stopped consuming alcohol and experienced any of the following symptoms during the period of abstinence: tremors, sweating, anxiety or agitation, seizures, confusion or disorientation, nausea and vomiting (outside cholera illness) suggestive of alcohol withdrawal.

### Vaccination and care-seeking behaviour

Only 15.2% of participants had been vaccinated during the 2021 OCV campaign, 7.3% had received the 2024 OCV. The majority (65.2%) reported no vaccination history for cholera. Regarding care-seeking behaviour, 65.7% of participants sought care for AWD), while 33.7% did not. Barriers to seeking care included, among others, financial constraints (27.0%), transport difficulties (19.7%), stigma from the community for having cholera (2.8%), lack of awareness of AWD risks (2.8%) and lack of community support (1%). Vaccination status and care-seeking behaviours are summarised in table 3.

**Table 3:** Vaccination status and care-seeking behaviour (n=178) of cholera-related deaths in communities during Zambia’s 2023–2024 outbreak

Characteristic	N	%
Vaccinated in 2021 OCV Campaign*		
Yes	27	15.2
No	116	65.2
Don't know	35	19.6
Vaccinated in 2024 OCV Campaign		
Yes	13	7.3
No	153	86.0
Don't know	12	6.7
Sought care for AWD		
Yes	117	65.7
No	60	33.7
Don't know	1	0.6
Considerations before seeking care (n=230 responses)†		
No issues		37.7
Financial constraints to support care		27.0
Lack of/difficulties accessing transport		19.7
Lack of family support		7.6
Fear of outcome at the facility		2.4
Stigma by the community for having Cholera		2.8
Lack of awareness of AWD risks		2.8
Lack of community support		1.0
Time to seek care (n=117)		
Less than 30 min	6	5.1
30 min to 1 hour	15	12.8
1 to 6 hours	42	35.9
6 to 24 hours	41	35.0
24 to 48 hours	8	6.8
2 to 5 days	5	4.3
First point of care (n=117)		
Primary health facility	60	51.3
First-level hospital	47	40.2
Retail pharmacy	3	2.6
Oral rehydration point	2	1.7
Private facility	2	1.7
Other‡	1	0.9
None	2	1.7
Received oral rehydration solution (ORS) at home		
Yes	75	42.13%
No	100	56.18%
Don’t know	3	1.69%
How long did it take to initiate ORS? (n=75)		
Within 30 min to 6 hours	58	37.91%
6 hours to 12 hours	25	16.34%
12 hours to 1 day	7	4.58%
More than 2 days	2	1.31%
1 day to 2 days	5	3.27%
Don’t know	56	36.60%

* Target population of 1 year and above in both campaigns.

† Each respondent could give more than one consideration taken before seeking care.

‡ Traditional healers; self-medication.

AWD, acute watery diarrhoea; OCV, oral cholera vaccines.

### Patterns in care-seeking behaviour and medication

Most of the deaths had not received ORS while at home (56.2%), with the majority (37.9%) initiating it within 30 min to 6 hours of symptom onset. Among those who sought care, the majority visited primary health facilities (PHFs) (51.3%) or first-level hospitals (40.2%). Delays in seeking care were notable, with only 5.1% accessing care within 30 min of symptom onset and 35.9% within 1–6 hours. Transportation to healthcare facilities was a challenge, notably en route among those who sought care. Private vehicles were the most used modality (28.2%), followed by walking (17.9%). Use of public vehicles (11.1%) and ambulances (4.3%) was limited. Furthermore, 36.8% of cases did not report a transport modality. Only 19.7% of participants who sought care reported taking medication for AWD, excluding ORS from the analysis. Antibiotics (31.4%) and painkillers (22.9%) were the most common treatments. Details on transportation used to reach health facilities and medication administered are provided in table 4.

**Table 4:** Transportation and medication details (n=117) of cholera-related deaths in communities during Zambia’s 2023–2024 outbreak

Characteristic	N	%
Transport modality		
Private vehicle	33	28.2
Walking	21	17.9
Public vehicle	13	11.1
Ambulance	5	4.3
Wheelbarrow	2	1.7
Not reported	43	36.8
Medication taken		
Yes	35	19.7
No	132	74.2
Don’t know	11	6.2
Type of medication (n=35)		
Antibiotics	12	34.3
Painkillers	9	25.7
Antibiotics and painkillers	4	11.4
Other (specify)*	4	11.4
Don't know	4	11.4
Painkillers and others	1	2.9
Traditional medication	1	2.9

* Activated charcoal, antimalarial, antiemetic, zinc.

## Discussion

The findings of this study highlight critical gaps and patterns in cholera-related mortality during the 2023–2024 outbreak in Zambia underscoring the complex challenges of managing cholera in resource-constrained settings. Overall, the study provided insights into demographic trends, environmental factors, clinical characteristics and care-seeking behaviour.

Most cholera-related deaths occurred among males, with the highest mortality observed in the economically active age group. This is consistent with a previous study conducted in Zimbabwe that demonstrated that the male sex was more vulnerable to community cholera death than the female sex.^16^ It has been postulated that the higher community deaths among males compared with females in Zambia may be associated in general with poor health-seeking behaviour among men.^17^ This pattern underscores the broader socioeconomic impact of cholera, particularly on households reliant on individuals in this age range for income generation. Furthermore, the proportion of deaths among children under the age of 5 reflects the increased vulnerability of this age group to diarrhoeal diseases, consistent with findings from other cholera-endemic regions.^418^,^20^ For example, in 2015, Ali* et al* estimated that cholera on average kills 91 000 people annually in endemic countries, with more than half of these being children under the age of 5.^4^ More recently, a study conducted in Bangladesh demonstrated the critical role of nutritional supplementation for children in the face of a cholera outbreak as malnourished children had fatal outcomes.^20^ This finding is in line with the present study which has shown that most cholera-related deaths were reported from households with smaller incomes. This highlights the importance of the relationship between nutrition and childhood mortalities.^21 22^ In contrast, limited educational attainment among the deceased, with having no formal education, may reflect a lack of awareness and understanding of the importance of adhering to preventive measures and the importance of seeking early care.^17 23^ The distribution of deaths across larger households, particularly those with five or more members, which make up over 45% of cases, raises concern about the potential for intrahousehold transmission and clusters of fatalities within families during the outbreak. These findings underscore the importance of targeted household-level interventions, including health education, rapid response teams and preventive measures (eg, household disinfection and contact vaccination) to break chains of transmission and prevent multiple deaths within the same family unit.^11 12^

The high proportion of deaths occurring at healthcare facilities on arrival suggests critical delays in both care-seeking and transport processes. This points to the patients either hesitating to seek medical care or encountering challenges in getting emergency transport services to a medical facility, resulting in undesired health outcomes, including death.^24 25^ Poor health-seeking behaviour could have been as a result of the cholera patients attempting to self-medicate or seek help away from conventional facilities as observed in other studies.^26 27^ Moreover, nearly 30% of deaths occurred at home. This further highlights the barriers to accessing healthcare services, including small household incomes, transportation challenges and the stigma associated with cholera.^28 29^ A substantial proportion of the deceased (over 60%) sought care at PHFs, indicating a general recognition within communities of the need for medical attention. However, the high proportion of deaths, despite contact with health services, further highlights potential challenges in both access to and quality of care.^28 29^

One plausible explanation is the severity of illness at presentation. Delays in seeking care due to financial constraints, lack of transport or low risk perception may have led patients to arrive at facilities in critical condition, with advanced dehydration or hypovolaemic shock, thus limiting the effectiveness of available interventions.^8 24 27^ Additionally, nearly 38% of patients received ORS within 6 hours of symptom onset, while 31% were administered antibiotics, pointing to variability in the timeliness and completeness of clinical response.

Importantly, these outcomes must also be interpreted in the context of health system strain. The large-scale nature of the outbreak likely overwhelmed PHFs, leading to bottlenecks in service delivery. Limited staffing, supply shortages, high patient volumes and logistical constraints may have impaired the system’s ability to provide rapid, standardised cholera care. Even when clinical protocols exist, their execution can falter under pressure, particularly in resource-limited settings.^9 27^

In addition, morning was the most common time of death, potentially reflecting the accumulation of untreated symptoms overnight, which could be addressed by strengthening community systems to access health services such as transport facilities and oral rehydration points (ORPs) which should be operated on a 24-hour basis.^24 30^ The delays in health-seeking likely contributed to the observed high mortality rates. Cholera requires rapid rehydration therapy to prevent fatal outcomes.^1^ Reliance on private vehicles and walking further highlights the limited availability of emergency transport services to resource-limited households. Promotion of the availability of emergency transport services within the community could mitigate these delays in care-seeking and response times.^28 30^

With regard to the clinical presentation of cholera, atypical symptoms including leg cramps, fever and headache were reported. This is in line with a study conducted by Mutale *et al* that reported atypical symptoms of cholera among the decedents they studied.^17^ The authors observed vulnerability of comorbidities including HIV/AIDS and hypertension to death, highlighting the health risks faced by individuals with pre-existing conditions during cholera outbreaks. This is in line with a recent study that established that patients with pre-existing conditions such as HIV and TB were more vulnerable to cholera and had poor outcomes.^8^ This finding emphasises the need for targeted interventions to address these vulnerabilities.^17^ Such interventions could include integrating cholera care into routine health services for people living with chronic illnesses.

Vaccination coverage was alarmingly low among the sample, with only 7.3% of participants vaccinated during the 2024 OCV campaign. The 2024 OCV campaign, conducted only in Lusaka district in January, had limited national coverage, with most cholera-related community deaths occurring before and during the campaign. However, low vaccination coverage reflects missed opportunities for preventive healthcare and suggests a need for established routine vaccinations prior to and during cholera seasons to underserved communities. The finding also highlights the need for focused community-based risk communication and community engagement, awareness campaigns and logistical planning to improve vaccination uptake in high-risk populations.^25 30^

Delayed health-seeking behaviour has been explored in other infectious disease outbreaks in our setting; hence, remedial factors have wider implications for improving patient outcomes.^31 32^ Future research could explore best practices to counter systemic barriers to care. These could include reducing distances to the facilities and aiding preference for self-treatments such as ORS in the community and placement of ORPs or Case Area Targeted Interventions to help reduce the number of community deaths. The Global Task Force on Cholera Control (GTFCC) recommends the prioritisation of community-driven approaches in cholera response strategies, as long-term success in reducing cholera’s impact relies on collaboration and effective use of community resources.^33^ Our study of community deaths during a cholera outbreak builds on the body of knowledge advocating for the decentralisation of care to break systemic barriers to treatment during an outbreak.

### Implications for public health

The study’s findings emphasise the need for integrated strategies to address cholera-related mortality. Public health efforts should prioritise:

- Strengthening community-based risk communication and community engagement to promote early care-seeking and dispel stigma.
- Enhancing primary healthcare infrastructure, including the availability of 24-hour services and emergency transport systems.
- Strengthening both the surge capacity and the routine readiness of front-line health facilities in cholera-prone areas.
- Enhancing early detection and referral mechanisms, decentralising case management and ensuring prepositioned supplies can reduce preventable mortality.
- Addressing socioeconomic barriers, such as financial constraints, through subsidies or community-based support systems.
- Expanding vaccination campaigns and ensuring equitable access to key populations.

### Limitations

While this study provides valuable insights, it is limited in its study design as it relied on retrospective data, which may be subject to recall bias. It is not possible to draw causal inference because of the study design. More studies with a prospective and analytical design are needed to draw causal inference.

The original survey sample included 209 individuals identified from the national cholera line list. However, the final analytic sample was reduced to 178 due to operational constraints; specifically, 25 entries lacked adequate contact information to enable follow-up, and 6 individuals declined participation. As these exclusions occurred prior to data collection and were unrelated to participant characteristics or outcomes, the potential for systematic bias is limited. Nevertheless, the absence of follow-up for some cases, particularly those without contact details, could under-represent individuals from marginalised or transient populations who may face greater barriers to care. Given the randomised nature of the original sample, the analytical cohort is still likely to reflect the broader population of interest, though some caution is warranted when generalising findings to all cholera-related deaths during the outbreak.

## Conclusions

Our study showed that delayed health-seeking behaviour, systemic barriers to accessing healthcare and low vaccination coverages contributed to the high cholera mortality rates in the 2023–2024 outbreak in Zambia. These findings highlight the crucial need for a multisectoral approach in addressing social determinants of health, improving vaccination coverage, establishing integrated community systems and structures to ease access to healthcare and investing in healthcare infrastructure. By prioritising the highlighted public health interventions and systemic reforms, the country can reduce cholera-related mortality and make communities more resilient against future cholera outbreaks and other public health threats.

## Supplementary material

10.1136/bmjopen-2025-102709 online supplemental file 1

## Data Availability

Data are available on reasonable request. Data may be obtained from a third party and are not publicly available.
