# Supplementary material for: Descriptive study of cholera-related deaths in communities during Zambia’s 2023–2024 outbreak: key insights
Source: BMJ Open. 2025 Oct 21;15(10):e102709. doi: 10.1136/bmjopen-2025-102709 (PMC12548604; doi:10.1136/bmjopen-2025-102709)
Supplement: online supplemental file 1 [file bmjopen-15-10-s001.docx]

**Questionnaire**

| **Cover sheet for questionnaire administered to relatives of the deceased** | | | | |
| --- | --- | --- | --- | --- |
| **People who participated in the interview** | | | | |
| ***Note:*** *A person who was there at the time of illness or death can participate in the interview****.*** *Up to four interviewees can be interviewed.* | | | | |
| **S.n** | **Name of the Interviewees** | **Relationship with the deceased** | **Was around at the time of:** | |
|  |  |  | **Illness** | **Death** |
| **1** |  |  | Yes No | Yes No |
| **2** |  |  | Yes No | Yes No |
| **3** |  |  | Yes No | Yes No |
| **4** |  |  | Yes No | Yes No |

| **SECTION A: Interview information** | | | | |
| --- | --- | --- | --- | --- |
|  | Interviewer name |  | | |
|  | Date of interview | DD/MM/YYYY / / / | | |
|  | Phone number of interviewer |  | | |
|  | ID Number/ Code for patient |  | | |
|  | Outcome of cholera infection | Death (case)  Recovery (control)  *If “Recovery” i.e. control was selected, skip to section B* | | |
|  | Interviewee name (Only for relatives/friends): |  | | |
|  | Relationship of interviewee with the deceased | _______________ | | |
|  | Was the interviewee around at the time of illness? | Yes  No | | |
|  | Was the interviewee around at the time of death | Yes  No | | |
|  | Date and time of death  **(only for deceased)** | DD/MM/YYYY / / /  Time (Hour: Minute) _ _ : _ _ | | |
|  | Place of death  **(only for deceased)** | Home/ Relatives’ Home (Name: )  Upon arrival at health facility  In transit from home to Health facility (________Kms)  In transit from health facility to health facility (________Kms) | | |
|  | | | | |
| **SECTION B. Background information of cholera patient** | | | | |
| **No** | **Questions** | | **Response** | |
|  | Age in years (if ≥2 years) | |  | |
|  | Age in months (if <2 years) | |  | |
|  | Sex | | Female  Male | |
|  | Place of residency | | Province District  Constituency Ward/ House number _ | |
|  | GPS coordinates | | __________________ | |
|  | Marital status | | Single  Married  Divorced  Widowed  Other (specify) _______________  Not applicable (e.g. minor) | |
|  | Religion | | Catholic  Protestant  Muslim  Other (specify) _______________ | |
|  | Educational status | | No formal Education  Primary  Secondary  Tertiary  Don’t know |  |
|  | Occupation | | Farmer  Merchant/trades person  Housewife  Daily laborer  Unemployed  Formal employment  Other (specify) ___________ | |
|  | Family’s monthly income if possible | | ZMW | |
|  | Vaccination for cholera | | Yes No Don’t know  *If No or Don’t know, skip to Section C.* | |
|  | If yes, then when was the last vaccination administered? | | DD/MM/YYYY / / / | |
| **SECTION C: Clinical presentation of cholera** | | | | |
|  | Diarrhoea | | Yes  No  Don’t know  *If No or Don’t know skip to question 15.* | |
|  | Date of onset of acute watery diarrhea (AWD) | | DD/MM/YYYY / / | |
|  | Vomiting | | Yes  No  Don’t know | |
|  | Leg cramps | | Yes  No  Don’t know | |
|  | Fever | | Yes  No  Don’t know | |
|  | Headache | | Yes  No  Don’t know | |
|  | Comorbid conditions/infections at the time of illness  **(multiple answers can be selected)** | | No comorbid condition  Hypertension  Diabetes mellitus  Heart disease  Malaria  HIV/AIDS  Tuberculosis  Malnutrition  Kidney disease  Others (specify) ________________  Don’t know | |
|  | If HIV Positive, on medications? | | Yes  No | |
|  | Is it known when the patient was started on ART? | | NOT STARTED  < 2 weeks  < 2months  < 6months  6 -12 months  >12months  Started but stopped (defaulted)  Not known | |
|  | Was the patient on any other medications prior to cholera for any of the listed conditions? | | Details: _________________________________________________ | |
|  |  | |  | |
| **SECTION D: Care at home (prior to seeking care)** | | | | |
|  | Received Oral Rehydration Solution (ORS) at home | | Yes  No  Don’t know  *If YES or Don’t know skip to question 23* | |
|  | Why had the patient not received ORS?  **(multiple answers can be selected)** | | Have not heard about ORS  Did not know that ORS can be used for cholera  No access to ORS sachets  No access to supplies to prepare home-made ORS  Did not like the taste  Belief that ORS does not help  Other (specify) ____________ | |
|  | If the patient had received ORS at home, how long did it take from the onset of symptoms to initiation of ORS? | | _______Days  _______Hours  _______Minutes | |
|  | What is needed to make ORS at home?  **(multiple answers can be selected)** | | Water  Sugar  Salt  Charcoal ash  Banana  Antibiotics  Other (specify) ___________________________  Don’t know | |
|  | Received antibiotics | | Yes No Don’t know | |
|  | Did the patient receive any of the following before seeking care | | Chlorine  Local brew (kachasu)  Charcoal ash  Bananas  Sour Milk  Other (specify) _____________ | |
| **SECTION E: Decision to seek early care (outside home)** | | | | |
|  | Was care sought for AWD? | | Yes  No  Don’t know | |
|  | How long did it take to seek care for AWD? | | _______ days  _______ hours | |
|  | Where did the patient go to seek care for AWD? **(Rank care seeking in chronological order) 1, 2, 3. ……….** | | Primary health care facility (HP, HC, CTU, CTC)  Private facility  Hospital  Retail pharmacy  Oral rehydration points (ORPs)  Traditional healers  Market /kiosk  Religious places of worship (Church/ mosque/ temple …etc) | |
|  | What issues did the patient or family have to consider prior to deciding to seek for early conventional/ standard medical care for AWD? | | No issues  Financial constraints to support care  Lack of family support  Lack of community support  Lack of access to community health structures (CBVs/CHWs/CHAs)  Lack of awareness of AWD risks (low risk perception)  Stigma by the community for having cholera  Fear of outcome at the facility  Others (specify) ____________________________________________________________________________________________________________________ | |
| **SECTION F: Standard medical care** | | | | |
|  | Was the patient treated for cholera in a health facility? | | Yes No Died on the way to the facility | |
|  | Name the health facility | | 1.____________________________________________________________  2. ____________________________________________________________  3._____________________________________________________________  4._____________________________________________________________ | |
|  | Referral information | | Self-referral from home to health facility  Referral from Cholera treatment Unit (CTU) to Cholera Treatment Centre (CTC) or first level hospital  Referral from Cholera Treatment Centre (CTC) to first level hospital  Referral from primary health care facility to Cholera Treatment Centre (CTC) or first level hospital  Referral from Oral Rehydration Point to Cholera Treatment Centre (CTC)  Other (specify)_______________________________________ | |
|  | What difficulties did the patient or family members have to reach health facility for AWD care? | | No difficulties  Financial constraints to afford transportation  Lack of transport  Long distance to the nearest health facility  Poor road access to health facility  Stigma and discrimination of AWD patients by transporters  Others (specify) ____________________________________________________________________________________________________________________ | |
|  | What transport modality was used to reach the health facility for AWD care? | | Ambulance Private vehicle Public vehicle Bicycle  Oxcart Wheelbarrow Walking  Other (specify) _____________________________ | |
|  | How long did it take to reach the health facility for AWD care? | | Died on the way  Less than 10 minutes  Between 10 mins to 1 hour  More than 1 hour to reach the facility  Not applicable | |
|  | What was the outcome of the first hospitalisation? | | Died on the way  Died within 1 hour of arrival at the facility  Died within 1 day  Patient was discharged successfully  Patient Left against medical advice  Others (specify) | |
|  | Following hospitalisation, what was the patient's status? | | Recovered fully  Diarrhoea continued, patient took meds at home  Diarrhoe continued, patient was taken to traditional healer  Diarrhoe continued, patient was taken to religious practitioner  Died at home following discharge  Patient was readmitted  Others (specify) | |
|  | What were the symptoms that led to the readmission? | | Vomiting  Diarrhoe  Weakness  Loss of Consciousness | |
|  | How long after the initial discharge did symptoms reappear? | | Persistent symptoms despite discharge  Started same day as discharge  Started 24-48hours after discharge  beyond 48hours following discharge | |
|  | If the patient was admitted, to which facility? | | Same facility  Different facility  Name of facility where readmitted: ___________________________ | |
|  | How many days after the initial discharge was the patient readmitted? | | Died on the way back home  Readmitted within 24 hours of discharge  Readmitted within 24-48hours following discharge  Readmitted beyond 48hours following discharge | |
|  | What was the patient’s final disposition following readmission? | | Died within 1 hour of arrival at the facility  Died within 1 day  Patient was discharged successfully  Patient Left against medical advice | |
| **SECTION G : Environmental conditions** | | | | |
|  | How many people live in the household? | | _______ | |
|  | Primary water source | | Piped water  Kiosk  Borehole  Shallow well  Other (specify) ________________________________________ | |
|  | How many household members **under 5 years** developed cholera at the time of illness of the patient  **(put 0 if none)** | | ______ | |
|  | How many household members **≥5 years** developed cholera at the time of illness of the patient  **(put 0 if none)** | | ______ | |
|  | How many of those listed in 48 and 49 above were did not visit a facility/notifiy the contact tracing team? | |  | |
|  | Type of toilet | | Pit latrine  Flush toilet  Open defecation  Other (specify) ________________________________________ | |
|  | How far is pit latrine from water source?  ***(Ask them to show you, so that you can estimate)*** | | ______ metres | |
|  | Household shares latrine | | Yes  No | |
|  | How many househoulds in the vicinity share the toilet listed in 51 above? | |  | |
|  |  | |  | |
| **SECTION H : Knowledge** | | | | |
|  | Heard of cholera before | | Yes  No | |
|  | Can cholera be prevented? | | Yes  No | |
|  | Can cholera be treated? | | Yes  No | |
|  | When someone experiences cholera symptoms when should they seek medical care at the facility? | | Immediately  Only when severe  Never | |
| **SECTION I: Behaviours** | | | | |
|  | Treated drinking water before illness | | Yes  No | |
|  | Treated drinking water today | | Yes  No | |
| **SECTION J: Quality of Care: To be filled for all patients who sought care in a health facility (Including CTCs and CTUs) by healthcare workers** | | | | |
|  | What was the date and time of arrival of the patient at the health facility? | | DD/MM/YYYY ____/____/______  Time (Hour: Minute) _ _ : _ _ | |
|  | How long did it take to clinically assess the patient after arrival at the facility? | | ______Hours, _____________Minutes | |
|  | Was the patient assessed for signs of dehydration at the triage area on arrival? | | Yes No Don’t know ( if no or Don’t know, skip to 53) | |
|  | Which of these danger signs of dehydration did the patient have on arrival at the health facility? | | No danger signs  Lethargic or unconscious  Absent or weak pulse  Respiratory distress | |
|  | What was the degree of dehydration of the patient on arrival at the health facility? | | Severe Dehydration Some dehydration No dehydration  Don’t know | |
|  | How long did it take to start rehydration therapy for the patient after arrival at the facility? | | ______Hours, _____________Minutes | |
|  | What type of treatment was given to the patient on arrival at the facility? | | Treatment with ORS  Intravenous rehydration  Antibiotics (Doxycycline, Tetracycline, Ciprofloxacin, Azithromycin, Or Erythromycin)  Other (specify):________________________________________ | |
|  | How long was the patient admitted to the inpatient area of the health facility or CTU/CTC? | | Not admitted  Less than 24 Hours (_______________ Hours)  More than 24 Hours (________________days) | |
|  | Were there challenges to provide clinical care during referral of the patient to the next level of care? | | No challenges  Pre-referal management was inadequate or suboptimal  Intrareferral manamagent was suboptimal  Transportation challenges during referral  Others (specify) __________________________________  No referal paper was attached | |
|  | What problems were met during cholera case management of the patient? | | The health provider was not trained on cholera case managment  The health providers was overstreched in managing other patients as well  Cholera case management was not optimally monitored  Cholera case management protocol was not used  Job aids for cholera case management were not avalable during the care provision  Lack of I.V fluids (Ringers lactate and Normal Saline)  Lack of Antibiotics for cholera treatment  Others (specify) ­­­­­­­__________________________________ | |
|  | Treatment plan provided | | A  B  C | |

**END OF QUESTIONNAIRE**

**THANK YOU**

**Appendix 2: Informed Consent Form**

**Survey Title: An Assessment of Cholera Related Community Deaths: 2023 – 2024 Cholera Outbreak in Zambia**

**Principal Investigator:**

**IRB No.: …………………..**

**PI Version, Date: Version 1.1, 29th May 2024**

**The Ministry of Health of Zambia through the Zambia National Public Health Institute is undertaking the An Assessment of Cholera Related Community Deaths: 2023 – 2024 Cholera Outbreak in Zambia. The information collected will help us better understand the factors associated with cholera related community deaths and guide policy on the fight against the disease. You are being asked to participate in this research study because you resident in Lusaka district.**

**If you agree to participate in this study, I or another member of my team will ask you questions about your current and past health in addition to simple questions about your age, sex, and signs and symptoms of cholera. The process will take 20 minutes.**

**The data collected from you during this study are important to science and public health. You will receive no financial benefit from the use of your data, but you can request for the data to be destroyed at any time.**

**We are not collecting any samples for this interview but only collecting the information that you provide during the interview.**

**There are no benefits to you for participating in this study. However, your participation will help us better understand the factors associated with cholera community deaths in Zambia and this will guide policy direction in the fight against this disease.**

**The results of this study will be presented to the Ministry of Health of Zambia. We will also attempt to keep members of your community informed about the study results.**

**Your participation in this investigation is voluntary. Refusal to take part will not result in any penalty or loss of services to which you are otherwise entitled. If you decide to take part, you are still free to withdraw at any time without penalty or loss of services and without giving a reason for your withdrawal. You may choose not to answer particular questions. If there is anything that you would prefer not to discuss, please feel free to say so. However, please note that the information you provide will improve our understanding of the outbreak and help us to prevent further transmission.**

**Please contact the field coordinator with the contact information provided below if you have any questions or concerns about this study. The coordinating investigator may be contacted using the following information:**

**Investigator:**

**Address: Zambia National Public Health Institute,**

**Tel:**

**Email:**

**If you have questions about your rights in regard to being part of this study, if you feel like you have not been treated fairly, or if you have other concerns you may contact the Principal Investigator using the following information:**

**The Chaiperson:**

**University of Zambia**

**Biomedical Research Ethics Committee**

**Ridgeway Campus**

**P O Box 50110**

**Lusaka, Zambia**

**Tel:**

**Email: unzarec@unza.zm**

**If all your questions have been satisfactorily answered, do you consent to answering a questionnaire?**

**Yes, I have read or listened to this information sheet for the study.**

**Yes, I understand that my participation is voluntary.**

**Yes, I consent to answering the questionnaire**

**Yes, the information obtained about me may be used in Scientific**

**Publication**

**Name of Participant: ……………………………………………………………………**

**Participant’s signature/thumbprint: ……………………Consent Date: ……………….**

**Witness’ signature/thumbprint: ………………………Date: …………………….........**

**Name of interviewer Conducting Informed Consent (Printed):**

**…………………………………………………………………………………………..**

**Signature: ………………………………..………Date………………………………...**

**Appendix 2: Informed Assent Form (Minors)**

**Survey Title: An Assessment of Cholera Related Community Deaths: 2023 – 2024 Cholera Outbreak in Zambia.**

**Principal Investigator:**

**IRB No.: ………………….**

**PI Version, Date: Version 1.1, 29th May 2024**

**Are you younger than 18 years old?**

**If no, assent should not be taken.**

**If yes, proceed with reading the following:**

**Statement to be read to child in the presence of their parent or guardian:**

**A disease called cholera can make people sick. There is a vaccine that you may be given to help severe cholera. If you are found to have the disease will be sent to the CTC/CTU. We are conducting this study to understand why people died from cholera. We are conducting this study to understand what risk factors are associated with dying from cholera in the community.**

**If you agree to be interviewed, we will ask you some questions. The questions will take about 20 minutes. If you do not want to answer any question, you do not have to. If you want to stop at any time, tell us and we will stop.**

**Your parent/guardian have said that it would be all right for you to take part in this study, but it is your decision. If you decide to be in the study now, you are free to change your mind later. You can ask me any questions you want. We will keep all the information you give us, private.**

**If you sign or mark this paper, it means that you have understood what I have said and that you want to be in the study. If you don’t want to be in the study, don’t sign this paper. Being in the study is up to you, and no one will be upset if you don’t sign this paper or if you change your mind later.**

**The above statement has been read to the child and the child agrees to participate in the research project.**

**________________________ ____________________________ __________**

**Print Child’s Name Signature or Left Thumb Impression of Date**

**Child**

**_______________________________________________________ _________**

**Print name of Signature of Parent/Legal Guardian Date**

**Parent/Legal Guardian**

**Witness’ signature/thumbprint:________________________ Date: _______**

**________________________ _____________________________ __________**

**Name Person Obtaining Consent Signature of Person Obtaining Consent Date**
