# Supplementary material for: Descriptive study of cholera-related deaths in communities during Zambia’s 2023–2024 outbreak: key insights
Source: BMJ Open. 2025 Oct 21;15(10):e102709. doi: 10.1136/bmjopen-2025-102709 (PMC12548604; doi:10.1136/bmjopen-2025-102709)
Supplement: Supplementary file 2 [file bmjopen-2025-102709.reviewer_comments.pdf]

PEER REVIEW HISTORY

BMJ Open publishes all reviews undertaken for accepted manuscripts. Reviewers are asked to complete a checklist review form and are provided with free text boxes to elaborate on their assessment. These free text comments are reproduced below.

ARTICLE DETAILS

Title (Provisional)

A Descriptive study of Cholera-Related Deaths in Communities during Zambia's 2023-2024 Outbreak: Key Insights

Authors

Mweso, Oliver; Shula, Agatha; Sialubanje, Cephas; Chanda, Stephen Longa; Shinjeka, Thelma; Mwangilwa, Kelvin; Chirwa, Lusungu; Kabwe, Danny; Mwanza, Jonathan; Mbewe, Nyuma; Fwoloshi, Sombo; Gebregiorgis, Azmach; Shikanga, O-tipo; Mwale, Moses; Nyirenda, Muyereka; Chipimo, Peter; Lisulo, Peter; Sinyange, Nyambe; Kapina, Muzala; Mubemba, Benjamin; Mukumbuta, Nawa; Bakyaita, Nathan; Chilengi, Roma

---

VERSION 1 - REVIEW

**Reviewer**	**1**
**Name**	**Dewi, Ayu Putu Madri**
**Affiliation**	**Amsterdam UMC Location AMC, Epidemiology and Data Science**
**Date**	**09-Apr-2025**
**COI**	**None**

Manuscript Title:

Descriptive study of Cholera-Related Deaths in Communities during Zambia's 2023-2024 Outbreak: Key Insights

Dear authors,

I am an Open Science researcher, who has checked your manuscript for its adherence to a number of Open Science practices.

Below you find the checklist that I used to assess your manuscript for adherence to a number of these practices.

Based on this assessment, I would suggest the following refinements/additions:

1. Availability of a protocol: Please provide a (link to the) protocol.

2. Pre-registration: Please provide information on whether and where the study was pre-registered.

3. Open Materials: N/A

4. Open Data: OK (Survey items and data is supplied in form of Tables)

5. Open Code: Please provide (a link to) the code used for the analyses in this study.

6. Citations: Please provide citations for your codes.

7. Software and tools used are described: OK

8. Open access publication: OK

9. Reporting checklist provided: OK

10. Preprint: Please state explicitly whether and where the preprint of this study can be found.

**Reviewer**	**2**
**Name**	**Kitahara, Kei**
**Affiliation**	**Okayama University**
**Date**	**09-May-2025**
**COI**	**None**

This study is a descriptive epidemiological investigation that elucidates the characteristics of community-based deaths during the large-scale cholera outbreak in Zambia from 2023 to 2024. It provides important insights relevant to public health practice. Notably, the finding that many deaths occurred either at home or upon arrival at health facilities highlights the significance of delayed healthcare access and socioeconomic barriers. The study logically underscores the need for expanded vaccination coverage and strengthened community-based response. By focusing on the drivers of cholera-related deaths in high-incidence areas, and revealing the gap between actual case fatality rates (CFRs) and WHO-recommended targets, the study addresses a critical public health concern. From the perspective of timely dissemination, this reviewer supports the publication of the manuscript with minimal delay. However, the following minor revisions are necessary:

The effective sample size was slightly reduced from the original 209 to 178, which may affect representativeness. A brief assessment of potential bias due to missing data should be included.

Please provide supplementary information on the nationwide or regional implementation status of the 2021 and 2024 Oral Cholera Vaccine (OCV) campaigns in Zambia, including what percentage of the population was vaccinated.

Since many readers may not be Zambian, Tables 1 and 2 should be supplemented with comparative national demographic data for Zambia.

Prevalence data for such as HIV, malaria, and hypertension in Zambia should also be provided to contextualize the findings.

Reviewer 3

Name Ramamurthy , Thandavaryan

Affiliation ICMR-National Institute of Cholera and Enteric Diseases, National Institute of Cholera and Enteric Diseases

Date 20-May-2025

COI None

This retrospective study describes about the attributes of community deaths during a large cholera outbreak in Zambia. The study results generated and analysed based on response to the questionnaire by the responders. This study has included both suspected and confirmed cholera deaths in the community, enroute or upon arrival to a healthcare facility prior to admission.

The study design is relied on retrospective data, which may recall bias.

Discussion points are all about known facts, that have been established in many investigations

Sentence on Page 4, line 59) and page 5, line 3 needs a reference.

Page 13, lines 34. Specific age group that used oral cholera vaccination is not clear.

Some of the points not discussed in detail include

Table 3 shows more than 60% of the patients used point of care facility (PCF). This raises several questions about the performance of the PCF, including available facilities, experienced staff, lack of proper case management etc.

Almost 38% of the patients received ORS support within 30 min after the onset of cholera and 31% used antibiotics (generic name not mentioned). Again, this gives background information about how the cholera cases were handled.

Information regarding deaths in the same family during the outbreak is not mentioned.

VERSION 1 - AUTHOR RESPONSE

Reviewer: 1
Ms. Ayu Putu Madri Dewi, Amsterdam UMC Location AMC
Comments to the Author:
Manuscript Title:

Descriptive study of Cholera-Related Deaths in Communities during Zambia's 2023-2024 Outbreak: Key Insights

Dear authors,
I am an Open Science researcher, who has checked your manuscript for its adherence to a number of Open Science practices.
Below you find the checklist that I used to assess your manuscript for adherence to a number of these practices.
Response: Thank you!
Based on this assessment, I would suggest the following refinements/additions:
1. Availability of a protocol: Please provide a (link to the) protocol.

Response: The protocol has been submitted with attached revisions. However, a link to the protocol and other analytical data may be obtained through a third party and are not publicly available or through a formal reasonable request to the Ministry of Health (MoH), info@moh.gov.zm, of the Government of the Republic of Zambia (GRZ) via the corresponding author.
2. Pre-registration: Please provide information on whether and where the study was pre-registered.

Response: The study was not pre-registered.
3. Open Materials: N/A
4. Open Data: OK (Survey items and data is supplied in form of Tables)
5. Open Code: Please provide (a link to) the code used for the analyses in this study.

Response: A link to the code and other analytical data may be obtained through a third party and are not publicly available or through a formal reasonable request to the Ministry of Health (MoH), info@moh.gov.zm, of the Government of the Republic of Zambia (GRZ) via the corresponding author.
6. Citations: Please provide citations for your codes.

Response: A link to the code and other analytical data with citation for codes may be obtained through a third party and are not publicly available or through a formal reasonable request to the Ministry of Health (MoH), info@moh.gov.zm, of the Government of the Republic of Zambia (GRZ) via the corresponding author.

7. Software and tools used are described: OK
8. Open access publication: OK
9. Reporting checklist provided: OK
10. Preprint: Please state explicitly whether and where the preprint of this study can be found.
Response: There is no current preprint. However, consideration has been given to submit to medRxiv.

Reviewer: 2
Dr. Kei Kitahara, Okayama University
Comments to the Author:
This study is a descriptive epidemiological investigation that elucidates the characteristics of community-based deaths during the large-scale cholera outbreak in Zambia from 2023 to 2024. It provides important insights relevant to public health practice. Notably, the finding that many deaths occurred either at home or upon arrival at health facilities highlights the significance of delayed healthcare access and socioeconomic barriers. The study logically underscores the need for expanded vaccination coverage and strengthened community-based response. By focusing on the drivers of cholera-related deaths in high-incidence areas, and revealing the gap between actual case fatality rates (CFRs) and WHO-recommended targets, the study addresses a critical public health concern. From the perspective of timely dissemination, this reviewer supports the publication of the manuscript with minimal delay. However, the following minor revisions are necessary:
Response: Thank you!
The effective sample size was slightly reduced from the original 209 to 178, which may affect representativeness. A brief assessment of potential bias due to missing data should be included.
Response: A brief assessment of potential bias has been included in the discussion section of the article in the paragraph detailing limitations: It reads 'The original survey sample included 209 individuals identified from the national cholera line list. However, the final analytic sample was reduced to 178 due to operational constraints; specifically, 25 entries lacked adequate contact information to enable follow-up, and six individuals declined participation. As these exclusions occurred prior to data collection and were unrelated to participant characteristics or outcomes, the potential for systematic bias is limited. Nevertheless, the absence of follow-up for some cases particularly those without contact details could underrepresent individuals from marginalized or transient populations who may face greater barriers to care. Given the randomized nature of the original sample, the analytic cohort is still likely to reflect the broader population of interest, though some caution is warranted when generalizing findings to all cholera-related deaths during the outbreak.'
Please provide supplementary information on the nationwide or regional implementation status of the 2021 and 2024 Oral Cholera Vaccine (OCV) campaigns in Zambia, including what percentage of the population was vaccinated.
Response: Supplementary information has been provided under Supplementary materials submitted with the article.2021 OCV campaign, 2024 OCV campaign
Since many readers may not be Zambian, Tables 1 and 2 should be supplemented with comparative national demographic data for Zambia.
Response: This article adds to the developing epidemiological profile of prevalence among deaths attributed to cholera in **communities** in Zambia. The comparative **national**

demographic profile for this is currently in development and not yet available for public access**.**
Prevalence data for such as HIV, malaria, and hypertension in Zambia should also be provided to contextualize the findings.
Response: This article adds to the developing epidemiological profile of prevalence data for HIV, Malaria and Hypertension among deaths attributed to cholera in **communities** in Zambia. The comparative **national** demographic profile for this is currently in development and not yet available for public access.

Reviewer: 3
Dr. Thandavaryan Ramamurthy, ICMR-National Institute of Cholera and Enteric Diseases
Comments to the Author:
This retrospective study describes about the attributes of community deaths during a large cholera outbreak in Zambia. The study results generated and analysed based on response to the questionnaire by the responders. This study has included both suspected and confirmed cholera deaths in the community, enroute or upon arrival to a healthcare facility prior to admission.

The study design is relied on retrospective data, which may recall bias.

Discussion points are all about known facts, that have been established in many investigations
Response: Thank you!

Sentence on Page 4, line 59) and page 5, line 3 needs a reference.
Response: References attached for both sentences.
Page 13, lines 34. Specific age group that used oral cholera vaccination is not clear.
Response: Age group specified as one and above in both campaigns (footnote, Table 3)
Some of the points not discussed in detail include

Table 3 shows more than 60% of the patients used point of care facility (PCF). This raises several questions about the performance of the PCF, including available facilities, experienced staff, lack of proper case management etc.

Almost 38% of the patients received ORS support within 30 min after the onset of cholera and 31% used antibiotics (generic name not mentioned). Again, this gives background information about how the cholera cases were handled.
Response: Discussion points have been included and read: 'A substantial proportion of the deceased (over 60%) sought care at Primary Health Facilities (PHFs), indicating a general recognition within communities of the need for medical attention. However, the high

proportion of deaths, despite contact with health services, highlights potential challenges in both access to and quality of care.

One plausible explanation is the severity of illness at presentation. Delays in seeking care due to financial constraints, lack of transport, or low risk perception may have led patients to arrive at facilities in critical condition, with advanced dehydration or hypovolemic shock, thus limiting the effectiveness of available interventions. Additionally, nearly 38% of patients received ORS within six hours of symptom onset, while 31% were administered antibiotics, pointing to variability in the timeliness and completeness of clinical response.

Importantly, these outcomes must also be interpreted in the context of health system strain. The large-scale nature of the outbreak likely overwhelmed PHFs, leading to bottlenecks in service delivery. Limited staffing, supply shortages, high patient volumes, and logistical constraints may have impaired the system's ability to provide rapid, standardized cholera care. Even when clinical protocols exist, their execution can falter under pressure, particularly in resource-limited settings.'

Information regarding deaths in the same family during the outbreak is not mentioned.

Response: Discussion points have been included and read: 'The distribution of deaths across larger households; particularly those with five or more members, which make up over 45% of cases, raises concern about the potential for intra-household transmission and clusters of fatalities within families during the outbreak. These findings underscore the importance of targeted household-level interventions, including health education, rapid response teams, and preventive measures (e.g., household disinfection and contact vaccination) to break chains of transmission and prevent multiple deaths within the same family unit'

Reviewer: 1

If you have selected 'Yes' above, please provide details of any competing interests.: Not applicable

Reviewer: 2

If you have selected 'Yes' above, please provide details of any competing interests.: Not applicable

Reviewer: 3

If you have selected 'Yes' above, please provide details of any competing interests.: Not applicable

---

VERSION 2 - REVIEW

**Reviewer**	**1**
**Name**	**Dewi, Ayu Putu Madri**

**Affiliation**	**Amsterdam UMC Location AMC, Epidemiology and Data Science**
**Date**	**23-Jul-2025**
**COI**	

As an open science researcher, I'd like to offer some feedback:

(1) Open data and code: The article indeed presents data in summary tabular form, I would argue it is not reproducible and not verifiable, as the raw data from the 209 samples has not been shared. That said, your informed consent form states that participants agreed their data could be used for scientific publication. Therefore, sharing anonymised data should not pose any ethical concerns. However, authors state that the data are included in the article or uploaded as supplementary information; but since this does not include the full set of 209 records, I would therefore suggest refinement by stating that the data and code are available upon request.

(2) Study protocol: Since ethical approval was obtained, a study protocol should be available. The article mentions, "The study protocol defined suspected cholera death...", which indicates a protocol exists. However, I find it problematic and is unclear how to verify it, as no such actual document is referenced. Since the protocol is not shared and as you said in the authors' responses letter that protocol only accessible via request, then I would recommend clarifying this by stating that the study protocol is not publicly available or is available upon request.

(3) Open materials: Adding the questionnaire is appreciated. OK.

VERSION 2 - AUTHOR RESPONSE

Ms. Ayu Putu Madri Dewi, Amsterdam UMC Location AMC

Comments to the Author:

As an open science researcher, I'd like to offer some feedback:

(1) Open data and code: The article indeed presents data in summary tabular form, I would argue it is not reproducible and not verifiable, as the raw data from the 209 samples has not been shared. That said, your informed consent form states that participants agreed their data could be used for scientific publication. Therefore, sharing anonymised data should not pose any ethical concerns. However, authors state that the data are included in the article or uploaded as supplementary information; but since this does not include the full set of 209 records, I would therefore suggest refinement by stating that the data and code are available upon request.

Response: Thank you! The statement that the data are included in the article or uploaded as supplementary information has been refined by stating that the data and code are available upon reasonable request. This is included in the article (page 25, line 18).

(2) Study protocol: Since ethical approval was obtained, a study protocol should be available. The article mentions, “The study protocol defined suspected cholera death...”, which indicates a protocol exists. However, I find it problematic and is unclear how to verify it, as no such actual document is referenced. Since the protocol is not shared and as you said in the authors' responses letter that protocol only accessible via request, then I would recommend clarifying this by stating that the study protocol is not publicly available or is available upon request.

Response: Thank you! A statement clarifying that the study protocol is available upon request has been included in the article (page 6, line 21).
